# Supplementary material for: Mapping domains of early life determinants of future multimorbidity across three UK longitudinal cohort studies
Source: Sci Rep. 2024 Sep 13;14:21454. doi: 10.1038/s41598-024-72275-5 (PMC11399113; doi:10.1038/s41598-024-72275-5)
Supplement: Supplementary file 1 — Supplementary Figure 1. [file 41598_2024_72275_MOESM1_ESM.pdf]

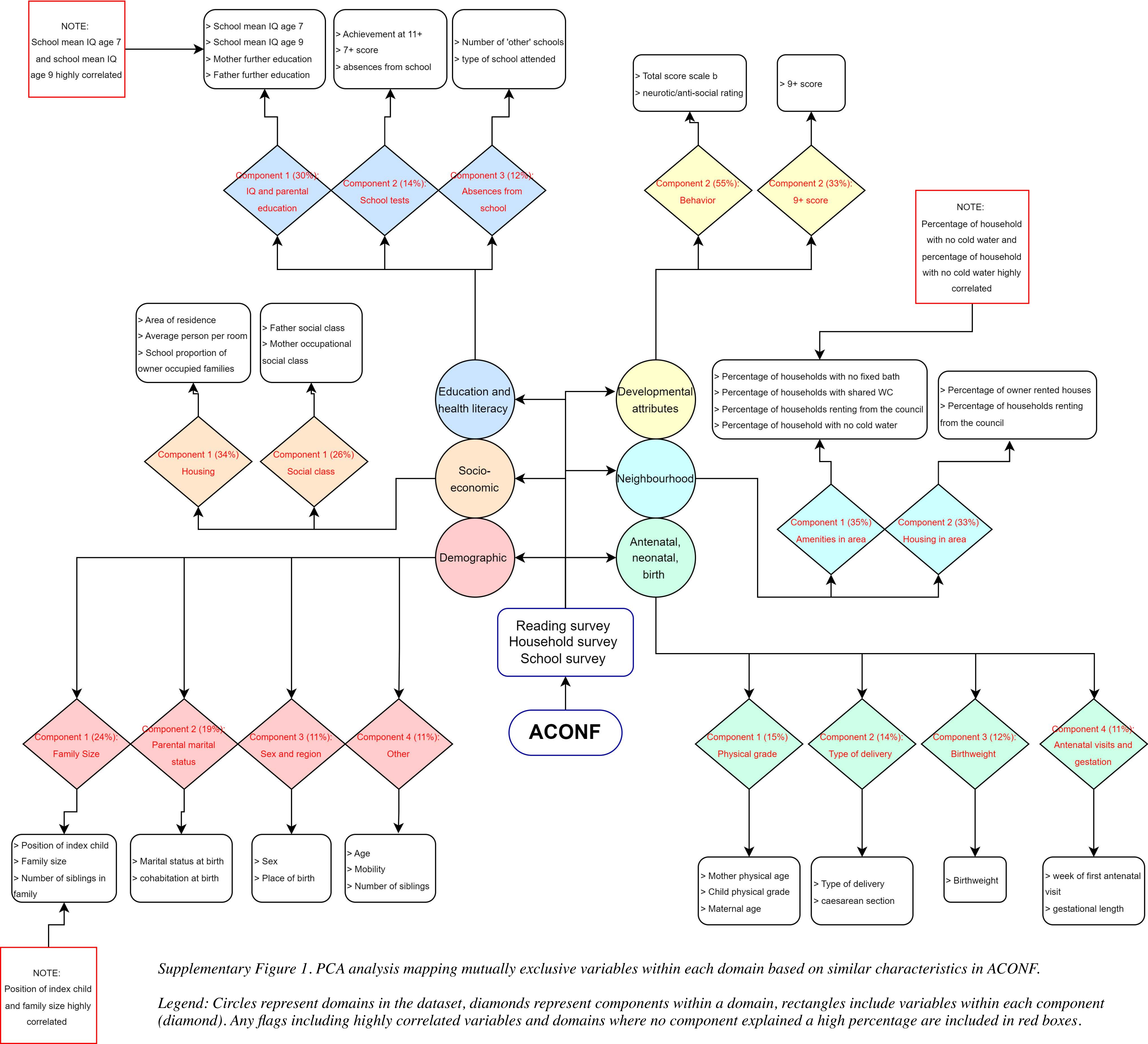

Supplementary Figure 1. PCA analysis mapping mutually exclusive variables within each domain based on similar characteristics in ACONF.

Legend: Circles represent domains in the dataset, diamonds represent components within a domain, rectangles include variables within each component (diamond). Any flags including highly correlated variables and domains where no component explained a high percentage are included in red boxes.
