# Supplementary material for: Mapping domains of early life determinants of future multimorbidity across three UK longitudinal cohort studies
Source: Sci Rep. 2024 Sep 13;14:21454. doi: 10.1038/s41598-024-72275-5 (PMC11399113; doi:10.1038/s41598-024-72275-5)
Supplement: Supplementary file 4 — Supplementary Information. [file 41598_2024_72275_MOESM4_ESM.pdf]

Supplementary Table 1. Variables identified from the data audit in BCS70

Variable	Description	Sweep of data collection	Supplementary variable	Domain
TeenMum	EVER A TEENAGE MOTHER (BD1AGEFB GROUPED)	Birth	No	Prenatal, antenatal, neonatal and birth
MatAge	AGE OF MOTHER AT CM'S BIRTH (FROM S1 VAR A0005A/S2 VAR E008)	Birth	No	Prenatal, antenatal, neonatal and birth
MatSmoke	SMOKING DURING PREGNANCY	Birth	No	Prenatal, antenatal, neonatal and birth
NoPrevPreg	NO OF PREVIOUS PREGNANCIES (ENTRIES ON FILE)	Birth	No	Prenatal, antenatal, neonatal and birth
Parity	PARITY	Birth	No	Prenatal, antenatal, neonatal and birth
NoAnteVisits	NUMBER OF ANTENATAL VISITS	Birth	No	Prenatal, antenatal, neonatal and birth
AnteInpVisit	OCCURRENCE OF ANTENATAL INPATIENT CARE	Birth	No	Prenatal, antenatal, neonatal and birth
PlaceDel	PLACE OF DELIVERY	Birth	No	Prenatal, antenatal, neonatal and birth
LabDur	DURATION 1ST STAGE OF LABOUR (HOURS	Birth	No	Prenatal, antenatal, neonatal and birth
MethodDel	METHOD OF DELIVERY	Birth	No	Prenatal, antenatal, neonatal and birth
Birthweight	BIRTHWEIGHT OF BABY IN GRAMS	Birth	No	Prenatal, antenatal, neonatal and birth
AnyOp	WERE ANY OPERATIONS PERFORMED	Birth	No	Prenatal, antenatal, neonatal and birth
Resus	BABY RESUSCITATION	Birth	No	Prenatal, antenatal, neonatal and birth
CongAbno	BORN WITH CONGENITAL ABNORMALITY	Birth	No	Prenatal, antenatal, neonatal and birth
Illness	BABY ILL AT BIRTH	Birth	No	Prenatal, antenatal, neonatal and birth
EverCongAbnor	CHILD EVER HAD ANY ABNORMALITY	10	No	Child health including check-ups and screening
EverBronchitis	CHILD EVER HAD BRONCHITIS	10	No	Child health including check-ups and screening
EverMeasles	EVER HAD MEASLES	10	No	Child health including check-ups and screening
EverMumps	EVER HAD MUMPS	10	No	Child health including check-ups and screening
EverWhooping	EVER HAD WHOOPING COUGH	10	No	Child health including check-ups and screening

EverChickenPox	EVER HAD CHICKEN POX	10	No	Child health including check-ups and screening
EverMeningitis	EVER HAD MENINGITIS	10	No	Child health including check-ups and screening
EverAcuteFever	EVER HAD OTHER ACUTE FEVER	10	No	Child health including check-ups and screening
EverTonsillectomy	EVER HAD TONSILLECTOMY	10	No	Child health including check-ups and screening
EverHerniaOp	EVER HAD HERNIA OPERATION	10	No	Child health including check-ups and screening
EverAppendicetomy	EVER HAD APPENDICECTOMY	10	No	Child health including check-ups and screening
EverOppSquint	EVER HAD OPERATION FOR SQUINT	10	No	Child health including check-ups and screening
EverVaccine	CHILD EVER HAD IMMUNISATION, VACCINATION	10	No	Child health including check-ups and screening
VacDiphtheria	VACCINATED AGAINST DIPHTHERIA	10	No	Child health including check-ups and screening
VacWhoopingCough	VACCINATED AGAINST WHOOPING COUGH	10	No	Child health including check-ups and screening
VacTetanus	VACCINATED AGAINST TETANUS	10	No	Child health including check-ups and screening
VacSmallpox	VACCINATED AGAINST SMALLPOX	10	No	Child health including check-ups and screening
VacPolio	VACCINATED AGAINST POLIOMYELITIS	10	No	Child health including check-ups and screening
VacMeasles	VACCINATED AGAINST MEASLES	10	No	Child health including check-ups and screening
VacTB	VACCINATED AGAINST TB (BCG)	10	No	Child health including check-ups and screening
VacOther	VACCINATED AGAINST OTHER	10	No	Child health including check-ups and screening
EverAdmHosp	HAS CHILD EVER BEEN ADMITTED TO HOSPITAL	10	No	Child health including check-ups and screening
TotalHospAdm	TOTAL NUMBER OF HOSPITAL ADMISSIONS	10	No	Child health including check-ups and screening
Outpatients	HAS CHILD EVER ATTENDED OUTPATIENTS?	10	No	Child health including check-ups and screening

TotalAccidents	TOTAL NUMBER OF ACCIDENTS	10	No	Child health including check-ups and screening
ChildSeenPsychiatrist	CHILD EVER BEEN TO CHILD PSYCHIATRIST	10	No	Child health including check-ups and screening
EmotionalBehaProb	HAS CHILD EVER HAD EMOTION,BEHAV. PROBS	10	No	Child health including check-ups and screening
IllnessHandicap	ANY EVIDENCE OF ANY ILLNESS/HANDICAP ETC	10	No	Child health including check-ups and screening
SoreThroat12m	RECURRENT SORE THROATS, PAST 12 MONTHS	10	No	Child health including check-ups and screening
MiddleEar12m	MIDDLE EAR INFECTION, PAST 12 MONTHS	10	No	Child health including check-ups and screening
HearingLoss12m	HEARING LOSS, PAST 12 MONTHS	10	No	Child health including check-ups and screening
Eczema12m	ECZEMA, PAST 12 MONTHS	10	No	Child health including check-ups and screening
HayFever12m	HAY FEVER, PAST 12 MONTHS	10	No	Child health including check-ups and screening
Asthma12m	ASTHMA, PAST 12 MONTHS	10	No	Child health including check-ups and screening
Wheezy12m	WHEEZY BRONCHITIS, PAST 12 MONTHS	10	No	Child health including check-ups and screening
Bronchitis12m	BRONCHITIS, PAST 12 MONTHS	10	No	Child health including check-ups and screening
Pneumonia12m	PNEUMONIA, PAST 12 MONTHS	10	No	Child health including check-ups and screening
HeartCond12m	PATHOLOGICAL HEART COND, PAST 12 MONTHS	10	No	Child health including check-ups and screening
AbdomPain12m	RECURRENT ABDOM PAIN, PAST 12 MONTHS	10	No	Child health including check-ups and screening
Hernia12m	INGUINAL HERNIA, PAST 12 MONTHS	10	No	Child health including check-ups and screening
UrinaryInf12m	URINARY INFECTION, PAST 12 MONTHS	10	No	Child health including check-ups and screening
OtherIllness12m	OTHER ILLNESSES, PAST 12 MONTHS	10	No	Child health including check-ups and screening
SystolicBP	BLOOD PRESSURE SYSTOLIC	10	No	Child health including check-ups and screening

DiastolicBP	BLOOD PRESSURE DIASTOLIC	10	No	Child health including check-ups and screening
AbnFancialGeneral	ABNORMAL FINDINGS A) FACIAL AND GENERAL	10	No	Child health including check-ups and screening
AbnSkin	ABNORMAL FINDINGS B) SKIN CONDITION	10	No	Child health including check-ups and screening
AbnENT	ABNORMAL FINDINGS C) ENT CONDITION	10	No	Child health including check-ups and screening
AbnUpperResp	ABNORMAL FINDINGS D) UPPER RESPIRATORY	10	No	Child health including check-ups and screening
AbnLowerResp	ABNORMAL FINDINGS E) LOWER RESPIRATORY	10	No	Child health including check-ups and screening
AbnCardiovasc	ABNORMAL FINDINGS F) CARDIOVASCULAR	10	No	Child health including check-ups and screening
AbnGastro	ABNORMAL FINDINGS G) GASTROINTESTINAL	10	No	Child health including check-ups and screening
AbnAbdoOther	ABNORMAL FINDINGS H) OTHER ABDOMINAL	10	No	Child health including check-ups and screening
AbnUrogenital	ABNORMAL FINDINGS I) UROGENITAL	10	No	Child health including check-ups and screening
AbnNeuro	ABNORMAL FINDINGS J) NEUROLOGICAL	10	No	Child health including check-ups and screening
AbnMusculo	ABNORMAL FINDINGS K) MUSCULO SKELETAL	10	No	Child health including check-ups and screening
AbnEndocrine	ABNORMAL FINDINGS L) ENDOCRINE	10	No	Child health including check-ups and screening
AbnBloodLymp	ABNORMAL FINDINGS M) BLOOD, LYMPHATIC	10	No	Child health including check-ups and screening
AbnMentalHandicap	ABNORMAL FINDINGS N) MENTAL HANDICAP	10	No	Child health including check-ups and screening
AbnBehEmot	ABNORMAL FINDINGS O) BEHAVIOUR/EMOTIONAL	10	No	Child health including check-ups and screening
CongAbnor	ANY EVIDENCE OF CONGENITAL ABNORMALITY	10	No	Child health including check-ups and screening
ChildBuild	DESCRIPTION OF CHILD'S BUILD	10	No	Child health including check-ups and screening
ConvReason	CONVULSION REASON	10	No	Child health including check-ups and screening

BMI	BMI	10	No	Child health including check-ups and screening
RutterBeh	TOTAL RUTTER BEHAVIOUR SCORE - GROUPED	10	No	Developmental attributes
EmotionProb	HAS CHILD EVER HAD EMOTION,BEHAV. PROBS	10	No	Developmental attributes
IllHandicap	ANY EVIDENCE OF ANY ILLNESS/HANDICAP ETC	10	No	Developmental attributes
ThrowCatchBall	THROWING BALL AND CATCHING	10	No	Developmental attributes
ThrowCatchClap	THROW BALL, CATCH BOTH HANDS, NO. CLAPS	10	No	Developmental attributes
StandRightLeg	STANDING ON RIGHT LEG, DID FOOT MOVE	10	No	Developmental attributes
StandLeftLeg	STANDING ON LEFT LEG, DID FOOT MOVE	10	No	Developmental attributes
WalkBackwards	WALKING BACKWARDS, NO. STEPS	10	No	Developmental attributes
Clumsy	DESCRIPTION OF CHILD'S COORDINATION	10	No	Developmental attributes
HandCoord	SCALE WORKS DEFTLY WITH HANDS	10	No	Developmental attributes
Temper	SCALE DISPLAYS OUTBURSTS OF TEMPER	10	No	Developmental attributes
ClumsyGame	SCALE CLUMSY AT GAMES	10	No	Developmental attributes
DiffKickBall	SCALE DIFFICULTY KICKING BALL	10	No	Developmental attributes
DiffPickingUp	SCALE DIFFICULTY PICKING UP SMALL OBJECTS	10	No	Developmental attributes
EdinbRead	EDINBURGH READING TEST SCORE (I3003 TO I3069)	10	No	Education and health literacy
EstReadAge	ESTIMATED READING AGE AT AGE 10 (DERIVED FROM ZB10READ AND AVERAGE AGE)	10	No	Education and health literacy
FriendlyMath	FRIENDLY MATHS TEST SCORE (I2504 TO I2575)	10	No	Education and health literacy
DiffMaths	DIFFICULTY WITH SCHOOL SUBS MATHS	10	No	Education and health literacy
DiffRead	READING	10	No	Education and health literacy
DiffWrite	WRITING	10	No	Education and health literacy
GenKnowledge	CHILD'S GENERAL KNOWLEDGE	10	No	Education and health literacy
IndepWork	DOES CHILD WORK INDEPENDENTLY	10	No	Education and health literacy
AbilityMaths	ABILITY IN MATHS	10	No	Education and health literacy
AbilityRead	ABILITY IN READING	10	No	Education and health literacy
AbilitySpell	ABILITY IN SPELLING	10	No	Education and health literacy

AbilityWrite	ABILITY IN CREATIVE WRITING	10	No	Education and health literacy
GoodSpell	GOOD AT SPELLING	10	No	Education and health literacy
TestGuess	TESTS ARE A LOT OF GUESS WORK	10	No	Education and health literacy
FatherQual	FATHERS ED QUALIFICATION	10	No	Education and health literacy
MotherQual	MOTHER ED QUALIFICATION	10	No	Education and health literacy
Region	STANDARD REGION OF RESIDENCE	10	No	Demographics
Sex	CHILD'S SEX	10	No	Demographics
HHNumber	NUMBER OF PERSONS IN HOUSEHOLD	10	No	Demographics
Parentalsep	CHILD LIVED WITH SAME PARENTS SINCE BORN	10	No	Demographics/ACE
ParentalDeath	REASON: DEATH OF A PARENT	10	No	Demographics/ACE
ChildEthnicity	ETHNIC GROUP STUDY CHILD 1	10	No	Demographics
MotherEthnicity	ETHNIC GROUP MOTHER 1	10	No	Demographics
FatherEthnicity	ETHNIC GROUP FATHER 1	10	No	Demographics
IllMother	SINCE CHILD 5YR ILLNESS IN FAMILY MOTHER	10	No	Transgenerational impact of parent health and health behaviours
IllFather	SINCE CHILD 5YR ILLNESS IN FAMILY FATHER	10	No	Transgenerational impact of parent health and health behaviours
MotherDrinkEarly	DID MOTHER DRINK DURING PREGNANCY, EARLY	10	No	Transgenerational impact of parent health and health behaviours
MotherDrinkLate	DID MOTHER DRINK DURING PREGNANCY, LATER	10	No	Transgenerational impact of parent health and health behaviours
MotherCough	COUGH: MOTHER	10	No	Transgenerational impact of parent health and health behaviours
FatherCough	COUGH: FATHER	10	No	Transgenerational impact of parent health and health behaviours
FatherSmoke	MOTHER'S PRESENT SMOKING HABITS	10	No	Transgenerational impact of parent health and health behaviours
MotherSmoke	FATHER'S PRESENT SMOKING HABITS	10	No	Transgenerational impact of parent health and health behaviours
MotherBMI	MOTHER BMI	10	No	Transgenerational impact of parent health and health behaviours
FatherBMI	FATHER BMI	10	No	Transgenerational impact of parent health and health behaviours
SocialClass	SOCIAL CLASS FROM FATHERS OCCUP (OR MOTHERS IF MISSING)	10	No	Socioeconomics

Benefits	RECEIVED STATE BENEFIT LAST 12 MONTHS? (C8.1 TO C8.11)	10	No	Socioeconomics
NumberHH	NUMBER OF PERSONS IN HOUSEHOLD	10	No	Socioeconomics
AccomType	WHAT ACCOMMODATION OCCUPIED BY FAMILY	10	No	Socioeconomics
HousingTenure	IS ACCOMMODATION OWNED OR RENTED?	10	No	Socioeconomics
UseBathroom	HAS FAMILY USE OF BATHROOM?	10	No	Socioeconomics
UseKitchen	HAS FAMILY USE OF KITCHEN?	10	No	Socioeconomics
Damp	IS ACCOMMODATION AFFECTED BY DAMP?	10	No	Socioeconomics
VanCar	DO YOU HAVE A CAR/VAN OF YOUR OWN	10	No	Socioeconomics
CouncilEstate	COUNCIL ESTATE	10	No	Socioeconomics
Income	AVERAGE INCOME	10	No	Socioeconomics
FatherEmploy	FATHER EMPLOYMENT STATUS	10	No	Socioeconomics
MotherEmploy	MOTHER EMPLOYMENT STATUS	10	No	Socioeconomics
MotherFig	RELATIONSHIP OF MOTHER FIGURE	10	No	Demographics/ACE
FatherFig	RELATIONSHIP OF FATHER FIGURE	10	No	Demographics/ACE
FatherManageChild	FATHER PLAYS ROLE IN MANAGING CHILD?	10	No	Parental family factors and parental ability to care for child
FamilyWalk	FAMILY ACTIVITIES A GO FOR WALKS	10	No	Parental family factors and parental ability to care for child
FamilyOutings	GO FOR OUTINGS TOGETHER	10	No	Parental family factors and parental ability to care for child
FamilyMeals	HAVE MEALS TOGETHER	10	No	Parental family factors and parental ability to care for child
FamilyHolidays	GO FOR HOLIDAYS TOGETHER	10	No	Parental family factors and parental ability to care for child
FamilyShopping	GO SHOPPING TOGETHER	10	No	Parental family factors and parental ability to care for child
FamilyChat	CHAT FOR AT LEAST 5 MINUTES	10	No	Parental family factors and parental ability to care for child
FamilyRestuarant	GO TO RESTAURANT TOGETHER	10	No	Parental family factors and parental ability to care for child
ParentMetTeach	PARENTS MET CHILD'S TEACHER	10	No	Parental family factors and parental ability to care for child

ParentSchLeaveAge	AGE CHILD WILL LEAVE SCHOOL	10	No	Parental family factors and parental ability to care for child
ParentsDiscussTeach	PARENTS' DISCUSSIONS WITH TEACHER	10	No	Parental family factors and parental ability to care for child
MotherIntrEd	MOTHER'S INTEREST IN CHILD'S EDUCATION	10	No	Parental family factors and parental ability to care for child
FatherIntrEd	FATHER'S INTEREST IN CHILD'S EDUCATION	10	No	Parental family factors and parental ability to care for child
MotherHostile	-MOTHER'S ATTITUDE HOSTILE	10	No	Parental family factors and parental ability to care for child
MotherDismissive	MOTHER'S ATTITUDE DISMISSIVE	10	No	Parental family factors and parental ability to care for child
FatherHostile	FATHER'S ATTITUDE HOSTILE	10	No	Parental family factors and parental ability to care for child
FatherDismissive	FATHER'S ATTITUDE DISMISSIVE	10	No	Parental family factors and parental ability to care for child
ParentsHearIdeas	PARENTS LIKE TO HEAR ABOUT IDEAS	10	No	Parental family factors and parental ability to care for child
FoolishTalkParents	FOOLISH TALKING TO PARENTS	10	No	Parental family factors and parental ability to care for child
EverCare	EVER BEEN IN CARE	10	No	ACE
GoToPark	GOES TO PARK/PLAYGROUND	10	No	Neighbourhood, physical environments and health care systems
CloseTraffic	CLOSENESS OF TRAFFIC TO HOUSE	10	No	Neighbourhood, physical environments and health care systems
RuralArea	NEIGHBOURHOOD DESCRIPTION - RURAL	10	No	Neighbourhood, physical environments and health care systems
PercCatchClosPackedHouse	AREA: OF CLOSELY PACKED HOUSES	10	No	Neighbourhood, physical environments and health care systems
PercCatchCouncilEst	AREA: COUNCIL ESTATE OF HOUSES	10	No	Neighbourhood, physical environments and health care systems
PercCatchLessExpHouse	AREA: LESS EXPENSIVE PRIVATE	10	No	Neighbourhood, physical environments and health care systems
PercCatchWellSpacedHouse	AREA: WELL-SPACED HOUSES	10	No	Neighbourhood, physical environments

				and health care systems
PercCatchLargeHouse	AREA: LARGE HOUSES SET IN OWN GROUNDS	10	No	Neighbourhood, physical environments and health care systems
PercCatchRural	AREA: MAINLY RURAL	10	No	Neighbourhood, physical environments and health care systems
PercCatchOther	AREA: OTHER TYPE OF NEIGHBOURHOOD	10	No	Neighbourhood, physical environments and health care systems
DescribeTrafficSchCathment	DESCRIPTION OF TRAFFIC	10	No	Neighbourhood, physical environments and health care systems
Area	CATCHMENT AREA DESCRIPTION	10	No	Neighbourhood, physical environments and health care systems
SpareTimeSport	SPARE TIME ACTIVITIES: A SPORTS	10	No	Health behaviours and diet
SpareTimeWalks	GOES FOR WALKS	10	No	Health behaviours and diet
SpareTimeSwimming	GOES SWIMMING	10	No	Health behaviours and diet
FamilyWalks	FAMILY ACTIVITIES A GO FOR WALKS	10	No	Health behaviours and diet
SportOutsideClass	HOURS SPORT-OUTSIDE PERIODS	10	No	Health behaviours and diet
TriedCig	HAVE YOU EVER TRIED A CIGARETTE	10	No	Health behaviours and diet
ChocSweetsOften	HOW OFTEN DO YOU EAT CHOCOLATE/SWEETS?	10	No	Health behaviours and diet
VegRel	VEGETARIAN BECAUSE OF RELIGIOUS REASONS	10	No	Religion, spirtuality and wider culture
RelBornInto	WHAT RELIGION WERE YOU BORN INTO?	10	No	Religion, spirtuality and wider culture
RelImport	IS RELIGION IMPORTANT PART OF YOUR LIFE?	10	No	Religion, spirtuality and wider culture
RelLucky	RELIGIOUS PEOPLE LUCKY TO HAVE BELIEFS	10	No	Religion, spirtuality and wider culture
RelValuedSoc	RELIGIOUS PEOPLE VALUED MEMBERS OF SOC.	10	No	Religion, spirtuality and wider culture
RelMisguided	RELIGIOUS PEOPLE ARE MISGUIDED	10	No	Religion, spirtuality and wider culture
RelHelp	RELIGIOUS PEOPLE HELP YOU IF IN TROUBLE	10	No	Religion, spirtuality and wider culture
NoAlcohRel	DON'T DRINK ALCOHOL - RELIGION FORBIDS	10	No	Religion, spirtuality and wider culture
DietRel	IS SPECIAL DIET FOR RELIGION/CULTURE?	10	No	Religion, spirtuality and wider culture
TimeSpentRel	TOTAL TIME SPENT: RELIGIOUS ACTIVITY	10	No	Religion, spirtuality and wider culture

Flag_Rel	FLAG FOR THOSE WHO REPORTED AT LEAST ONE QUESTION ON RELIGION	10	No	Religion, spirtuality and wider culture
MumAsthma5	NAT. MUM - ASTHMA	5	Yes	Transgenerational impact of parent health and health behaviours
MumHayfever5	NAT. MUM - HAYFEVER	5	Yes	Transgenerational impact of parent health and health behaviours
MumEczema5	NAT. MUM - ECZEMA	5	Yes	Transgenerational impact of parent health and health behaviours
MumLateReader5	NAT. MUM - LATE READER	5	Yes	Transgenerational impact of parent health and health behaviours
MumPoorReader5	NAT. MUM - POOR READER	5	Yes	Transgenerational impact of parent health and health behaviours
MumConvulsions5	NAT. MUM - CONVULSIONS	5	Yes	Transgenerational impact of parent health and health behaviours
MumLateSpeaker5	NAT. MUM - LATE SPEAKER	5	Yes	Transgenerational impact of parent health and health behaviours
DadAsthma5	NAT. DAD - ASTHMA	5	Yes	Transgenerational impact of parent health and health behaviours
DadHayfever5	NAT. DAD - HAYFEVER	5	Yes	Transgenerational impact of parent health and health behaviours
DadEczema5	NAT. DAD - ECZEMA	5	Yes	Transgenerational impact of parent health and health behaviours
DadLateReader5	NAT. DAD - LATE READER	5	Yes	Transgenerational impact of parent health and health behaviours
DadPoorReader5	NAT. DAD - POOR READER	5	Yes	Transgenerational impact of parent health and health behaviours
DadConvulsions5	NAT. DAD - CONVULSIONS	5	Yes	Transgenerational impact of parent health and health behaviours
DadLateSpeaker5	NAT. DAD - LATE SPEAKER	5	Yes	Transgenerational impact of parent health and health behaviours
MotherSmoke5	SMOKING HABIT OF MOTHER	5	Yes	Transgenerational impact of parent health and health behaviours
FatherSmoke5	SMOKING HABIT OF FATHER	5	Yes	Transgenerational impact of parent health and health behaviours
RelwithNeigh5	RELATIONSHIP OF FAMILY WITH NEIGHBOURS	5	Yes	Neighbourhood, physical environments and health care systems
SocRatingNeigh5	SOCIAL RATING OF NEIGHBOURHOOD	5	Yes	Neighbourhood, physical environments

				and health care systems
MaternalMalaise5	TOTAL MALAISE SCORE - GROUPED IN STANDARD WAY	5	Yes	Transgenerational impact of parent health and health behaviours/ACE
NoHealthVisits5	TOTAL HV VISITS SINCE BIRTH	5	Yes	Child health including check-ups and screening
NoCHCVisits5	TOTAL CHC ATTENDANCES SINCE BIRTH	5	Yes	Child health including check-ups and screening
ScreeningPKU5	ANY SCREENING FOR PKU LOGGED ON HV OR CHC RECORDS?	5	Yes	Child health including check-ups and screening
ScreeningCDH5	ANY SCREENING FOR CDH (HIP) LOGGED ON HV OR CHC RECORDS?	5	Yes	Child health including check-ups and screening
ScreeningHearing5	ANY SCREENING FOR HEARING LOGGED ON HV OR CHC RECORDS?	5	Yes	Child health including check-ups and screening
ScreeningSquint5	ANY SCREENING FOR SQUINT LOGGED ON HV OR CHC RECORDS?	5	Yes	Child health including check-ups and screening
ScreeningVision5	ANY SCREENING FOR VISION LOGGED ON HV OR CHC RECORDS?	5	Yes	Child health including check-ups and screening
TotalNoScreenings5	TOTAL NUMBER OF SCREENINGS FOR GENERAL DEVELOPMENT, HEARING, VISION	5	Yes	Child health including check-ups and screening
AnyEatProb5	EATING OR APPETITE PROBLEMS	5	Yes	Health behaviours and diet
EatProbUnderEat5	NOT EATING ENOUGH	5	Yes	Health behaviours and diet
EatProbOvereating5	OVEREATING	5	Yes	Health behaviours and diet
EatProbFaddiness5	FADDINESS	5	Yes	Health behaviours and diet
OtherEatingProb5	OTHER EATING PROBLEM	5	Yes	Health behaviours and diet
KeepSport16	I AM KEEN ON SPORTS	16	Yes	Health behaviours and diet
PlaySportClub16	LEISURE: PLAY SPORTS (AT CLUB/CENTRE ETC)	16	Yes	Health behaviours and diet
PlaySportStr~16	LEISURE: PLAY SPORTS (STREET/PARK/ETC)	16	Yes	Health behaviours and diet
IntPeopleDif~16	INTERESTED IN PEOPLE DIFF. RACE-RELIGION	16	Yes	Religion, spirtuality and wider culture
RelEdEssenti~16	RELIGIOUS EDUC. IS ESSENTIAL IN SCHOOLS	16	Yes	Religion, spirtuality and wider culture
NoCigs16	HOW MANY CIGARETTES DO YOU SMOKE A WEEK	16	Yes	Health behaviours and diet
SmokingHabits16	DESCRIPTION SMOKING/NON SMOKING HABITS	16	Yes	Health behaviours and diet

AlcoholPastW~16	NUMBER OF DAYS HAD ALCOHOL PAST WEEK	16	Yes	Health behaviours and diet
TotalAlcUnits16	TOTAL UNITS OF ALCOHOL IN PAST WEEK	16	Yes	Health behaviours and diet
VegReligion16	VEGETARIAN BECAUSE OF RELIGIOUS REASONS	16	Yes	Religion, spirtuality and wider culture
ParentHitYou16	WHICH PARENT HIT YOU?	16	Yes	ACE
RelBornInto16	WHAT RELIGION WERE YOU BORN INTO?	16	Yes	Religion, spirtuality and wider culture
RelImportant16	IS RELIGION IMPORTANT PART OF YOUR LIFE?	16	Yes	Religion, spirtuality and wider culture
RelLuckyBeleifs16	RELIGIOUS PEOPLE LUCKY TO HAVE BELIEFS	16	Yes	Religion, spirtuality and wider culture
RelOldFashioned16	RELIGIOUS PEOPLE ARE OLD FASHIONED	16	Yes	Religion, spirtuality and wider culture
RelValuedSoc16	RELIGIOUS PEOPLE VALUED MEMBERS OF SOC.	16	Yes	Religion, spirtuality and wider culture
RelMisguided16	RELIGIOUS PEOPLE ARE MISGUIDED	16	Yes	Religion, spirtuality and wider culture
RelHelpTrouble16	RELIGIOUS PEOPLE HELP YOU IF IN TROUBLE	16	Yes	Religion, spirtuality and wider culture
RelNoDiff16	RELIGIOUS PEOPLE NO DIFFERENT FROM REST	16	Yes	Religion, spirtuality and wider culture
NoCig16	NUMBER OF CIGARETTES SMOKED IN A WEEK	16	Yes	Health behaviours and diet
NoisyNeighbours16	IN AREA NOISY NEIGHBOURS OR LOUD PARTIES	16	Yes	Neighbourhood, physical environments and health care systems
Grafiti16	IN AREA GRAFFITI ON WALLS OR BUILDINGS	16	Yes	Neighbourhood, physical environments and health care systems
Teenagersstreet16	IN AREA TEENAGERS HANGING ROUND STREETS	16	Yes	Neighbourhood, physical environments and health care systems
DrunksStreet16	IN AREA DRUNKS OR TRAMPS ON STREETS	16	Yes	Neighbourhood, physical environments and health care systems
RubbishStreet16	IN AREA LOTS OF RUBBISH LYING ABOUT	16	Yes	Neighbourhood, physical environments and health care systems
UnfairlyTreaTRel16	UNFAIRLY TREATED BECAUSE OF RELIGION	16	Yes	Religion, spirtuality and wider culture
U10SexualApproach16	UNDER 10YRS WHEN SEXUAL APPROACH MADE	16	Yes	ACE
SexualAppr10_15	AGED 10-15YRS WHEN SEXUAL APPROACH MADE	16	Yes	ACE
SexualApproach1yr16	SEXUAL APPROACH MADE IN PAST YEAR	16	Yes	ACE

NoSexualApproach1yr16	NO. TIMES PAST YR UNWELCOME SEX APPROACH	16	Yes	ACE
NoAlcoholRel16	DON'T DRINK ALCOHOL - RELIGION FORBIDS	16	Yes	Religion, spirtuality and wider culture
HealthyDiet16	KNOW HOW TO GET A HEALTHY DIET	16	Yes	Health behaviours and diet
SportCentreFarAway16	SPORT-COMMUNITY CENTRE TOO FAR AWAY	16	Yes	Neighbourhood, physical environments and health care systems
ActReligion16	OTHER ACTIV. CONNECTED TO YOUR RELIGION	16	Yes	Religion, spirtuality and wider culture
MotherIllness16	MOTHER ILLNESS OR DISABLEMENT SINCE 10YR	16	Yes	Transgenerational impact of parent health and health behaviours
FatherIllness16	FATHER ILLNESS OR DISABLEMENT SINCE 10YR	16	Yes	Transgenerational impact of parent health and health behaviours
HusbandSmoke16	DOES HUSBAND SMOKE AT ALL?	16	Yes	Transgenerational impact of parent health and health behaviours
MotherSmoke16	SELF (MOTHER) SMOKE AT ALL?	16	Yes	Transgenerational impact of parent health and health behaviours
DescNeighbourhood16	DESCRIPTION OF NEIGHBOURHOOD	16	Yes	Neighbourhood, physical environments and health care systems
CMEatPeasGreens16	TEENAGER EATS PEAS/GREEN BEANS	16	Yes	Health behaviours and diet
CMEatOtherGreens16	TEENAGER EATS OTHER GREEN VEGATABLES	16	Yes	Health behaviours and diet
CMEatRootVeg16	TEENAGER EATS ROOT VEGATABLES	16	Yes	Health behaviours and diet
CMEatGreenSalad16	TEENAGER EATS GREEN SALAD	16	Yes	Health behaviours and diet
CMEatFruit16	TEENAGER EATS FRESH FRUIT	16	Yes	Health behaviours and diet
DietForReligion16	IS SPECIAL DIET FOR RELIGION/CULTURE?	16	Yes	Religion, spirtuality and wider culture
CMAlcohol16	HOW OFTEN TEENAGER HAS ALCOHOLIC DRINK	16	Yes	Health behaviours and diet
FatherAlcohol16	HOW OFTEN HUSBAND HAS ALCOHOLIC DRINK	16	Yes	Health behaviours and diet
MotherAlcohol16	HOW OFTEN MOTHER HAS ALCOHOLIC DRINK	16	Yes	Health behaviours and diet
CMStayHealthy16	DOES TEENAGER DO THINGS TO KEEP HEALTHY?	16	Yes	Health behaviours and diet
MotherStayHealthy16	DOES MOTHER DO THINGS TO KEEP HEALTHY?	16	Yes	Health behaviours and diet
HusbandStayHealthy16	DOES HUSBAND DO THINGS TO KEEP HEALTHY?	16	Yes	Health behaviours and diet

TriedSolvents16	EVER TRIED SNIFFING GLUE/SOLVENTS?	16	Yes	Health behaviours and diet
TriedUppers16	HAVE YOU EVER TRIED TAKING UPPERS?	16	Yes	Health behaviours and diet
TriedDowners16	HAVE YOU EVER TRIED TAKING DOWNERS?	16	Yes	Health behaviours and diet
TriedCannabis16	HAVE YOU EVER TRIED TAKING CANNABIS?	16	Yes	Health behaviours and diet
TriedCocaine16	HAVE YOU EVER TRIED TAKING COCAINE?	16	Yes	Health behaviours and diet
TriedSemeron16	HAVE YOU EVER TRIED TAKING SEMERON?	16	Yes	Health behaviours and diet
TriedHeroin16	HAVE YOU EVER TRIED TAKING HEROIN?	16	Yes	Health behaviours and diet
Neighbourhood16	WHAT ARE PEOPLE LIKE IN NEIGHBOURHOOD?	16	Yes	Neighbourhood, physical environments and health care systems
WalkAloneDark16	EVER WALK ALONE IN YOUR AREA AFTER DARK?	16	Yes	Neighbourhood, physical environments and health care systems
SafeWalkAlone16	IF WALK ALONE HOW SAFE WOULD YOU FEEL?	16	Yes	Neighbourhood, physical environments and health care systems
DescArea16	DESCRIBE HOUSES-FLATS IN YOUR AREA	16	Yes	Neighbourhood, physical environments and health care systems
ParentalMalaise16	TOTAL MALAISE SCORE GROUPED (AGE 16)	16	Yes	Transgenerational impact of parent health and health behaviours
CMMalaise16	TOTAL MALAISE SCORE (22 QUESTIONS) GROUPED	16	Yes	Child health including check-ups and screening

All variables selected from the data audit in the BCS70 at birth, age 5, age 10 and age 16

All variables selected from the data audit in the BCS70 at birth, age 5, age 10 and age 16

Supplementary Table 2. Variables identified from the data audit in NCDS

Variable	Description	Sweep of data collection	Supplementary variable	Domain
ncdsid	SERIAL NUMBER	N/A	N/A	N/A
n0region	REGION AT PMS (1958) - BIRTH	Birth	No	Prenatal, antenatal, neonatal and birth

MatAge	MOTHER'S AGE LAST BIRTHDAY,IN YEARS	Birth	No	Prenatal, antenatal, neonatal and birth
Matweight	MOTHER'S WEIGHT IN STONES,1958	Birth	No	Prenatal, antenatal, neonatal and birth
AnteVisits	TOTAL NUMBER OF ANTENATAL VSITS	Birth	No	Prenatal, antenatal, neonatal and birth
MatSmoke	SMOKING DURING PREGNANCY	Birth	No	Prenatal, antenatal, neonatal and birth
Parity	PARITY	Birth	No	Prenatal, antenatal, neonatal and birth
LabDur	DURATION OF LABOUR-1ST STAGE:HOURS	Birth	No	Prenatal, antenatal, neonatal and birth
FoetDiss	FOETAL DISTRESS	Birth	No	Prenatal, antenatal, neonatal and birth
Birthweight	WEIGHT OF BABY IN OUNCES	Birth	No	Prenatal, antenatal, neonatal and birth
BirthSpace	INTERVAL BETWEEN THIS BIRTH AND LAST	Birth	No	Prenatal, antenatal, neonatal and birth
Breastfed	BREAST FED PARTIALLY OR WHOLLY	Birth	No	Prenatal, antenatal, neonatal and birth
POD	PLACE OF DELIVERY	Birth	No	Prenatal, antenatal, neonatal and birth
PLANC	PLACE OF ANTENATAL CARE	Birth	No	Prenatal, antenatal, neonatal and birth
ABNORM00	NO OBSTETRIC, PREGNANCY ABNORMALITY	Birth	No	Prenatal, antenatal, neonatal and birth
AD2HOSP	ADMISSION TO HOSPITAL	Birth	No	Prenatal, antenatal, neonatal and birth
RESUS	RESUSCITATION	Birth	No	Prenatal, antenatal, neonatal and birth
ILLNESS	BABY'S ILLNESS	Birth	No	Prenatal, antenatal, neonatal and birth
gmeasles11a	GERMAN MEASLES DERIVED VARIABLE	11	No	Child health including check-ups and screening
mumps11a	MUMPS DERIVED VARIABLE	11	No	Child health including check-ups and screening
chickenpox11a	CHICKEN POX DERIVED VARIABLE	11	No	Child health including check-ups and screening
whoopingc11a	WHOOPING COUGH DERIVED VARIABLE	11	No	Child health including check-ups and screening
scarletfever11a	SCARLET FEVER DERIVED VARIABLE	11	No	Child health including check-ups and screening
rheumaticfev11	RHEUMATIC FEVER DERIVED VARIABLE	11	No	Child health including check-ups and screening
hepatitis11	HEPATITIS DERIVED VARIABLE	11	No	Child health including check-ups and screening
meningitis11	MENINGITIS DERIVED VARIABLE	11	No	Child health including check-ups and screening
tb11	TB DERIVED VARIABLE	11	No	Child health including check-ups and screening
infecdis11a	INFECTIOUS DISEASE DERIVED VARIABLE	11	No	Child health including check-ups and screening
asthma11b	EVER ASTHMA	11	No	Child health including check-ups and screening
bronchitis11b	EVER WHEEZY BRONCHITIS	11	No	Child health including check-ups and screening
hayfever11	N PAST YEAR-ANY HAY FEVER, RHINITIS	11	No	Child health including check-ups and screening

recvomiting11	IN PAST YEAR-RECURRENT VOMITING	11	No	Child health including check-ups and screening
mouthulcers11	IN PAST YEAR-RECURRENT MOUTH ULCERS	11	No	Child health including check-ups and screening
throatinfec11a	RECURR THROAT,EAR INFECTS REQ TRTMENT	11	No	Child health including check-ups and screening
nephritis11	NEPHRITIS DERIVED VARIABLE	11	No	Child health including check-ups and screening
nephrosis11	NEPHROSIS DERIVED VARIABLE	11	No	Child health including check-ups and screening
urinaryinfec11	URINARY INFECTION DERIVED VARIABLE	11	No	Child health including check-ups and screening
poorbreathing11	POOR BREATHING DERIVED VARIABLE	11	No	Child health including check-ups and screening
manycolds11	MANY COLDS DERIVED VARIABLE	11	No	Child health including check-ups and screening
redeyes11	2 SORE, RED EYES DERIVED VARIABLE	11	No	Child health including check-ups and screening
coldhands11	VERY COLD HANDS DERIVED VARIABLE	11	No	Child health including check-ups and screening
epilepsy11	EPILEPY DERIVED VARIABLE	11	No	Child health including check-ups and screening
measles11	MEASLES COMPOSITE DERIVED VARIABLE	11	No	Child health including check-ups and screening
headache11y	HEADACHES COMPOSITE DERIVED VARIABLE	11	No	Child health including check-ups and screening
abpain11y	ABDOMINAL PAIN COMPOSITE DERIVED VARIABLE	11	No	Child health including check-ups and screening
heartcondition11y	HEART CONDITION COMPOSITE DERIVED VARIABLE	11	No	Child health including check-ups and screening
eczema11y	ECZEMA COMPOSITE DERIVED VARIABLE	11	No	Child health including check-ups and screening
hernia11y	HERNIA COMPOSITE DERIVED VARIABLE	11	No	Child health including check-ups and screening
tonsilitis11y	TONSILITIS COMPOSITE DERIVED VARIABLE	11	No	Child health including check-ups and screening
somaticsymptoms11y	SCALE TOTAL SOMATIC SYMPTOMS 2P	11	No	Child health including check-ups and screening
gastrosymptoms11y	SCALE TOTAL GASTROINTESTINAL SYMPTOMS 2P	11	No	Child health including check-ups and screening
gastroillness11y	SCALE TOTAL GASTROINTESTINAL ILLNESS 2P	11	No	Child health including check-ups and screening
longstandingIll11y	SCALE TOTAL LONGSTANDING ILLNESS 2P	11	No	Child health including check-ups and screening
atopy11y	SCALE TOTAL ATOPY 2P	11	No	Child health including check-ups and screening
infecillness11	SCALE TOTAL INFECTIOUS ILLNESS 2P	11	No	Child health including check-ups and screening
Hearing11	CHILD ALWAYS GOOD HEARING BOTH EARS	11	No	Child health including check-ups and screening
HospAd11	NO.OF TIMES CHLD ADMITTED TO HOSPITAL	11	No	Child health including check-ups and screening
ChildOutPatient	HAS CHILD BEEN AN OUTPATIENT	11	No	Child health including check-ups and screening

PsychTreatment	PSYCHIATRIC,PSYCHOLOGICAL TREATMENT	11	No	Child health including check-ups and screening
BSAGAnxiety	TOT SCORE-BSAG ANXIETY ACCEPTNCE,CHILDN	11	No	Child health including check-ups and screening
BSAGDepression	TOTAL SCORE-BSAG DEPRESSION SYNDROME	11	No	Child health including check-ups and screening
ChildIrritable	IS CHILD IRRITABLE,QUICK TEMPERED	11	No	Developmental attributes
ChildClumsy	ANY ABNORMALITY,CLUMSINESS-MC 1:4	11	No	Developmental attributes
WalkLine	WALKING BACKWARDS ALONG STRAIGHT LINE	11	No	Developmental attributes
StandRight	STANDING ON RIGHT FOOT 15 FOR SECONDS	11	No	Developmental attributes
StandLeft	STANDING ON LEFT FOOT FOR 15 SECONDS	11	No	Developmental attributes
HeelToe	STANDING HEEL TO TOE FOR 15 SECONDS	11	No	Developmental attributes
ChildHandControl	CHILD HAS POOR HAND CONTROL	11	No	Developmental attributes
ChildCoord	CHILD HAS POOR PHYSICAL CO-ORDINATION	11	No	Developmental attributes
InconsBeh	TOT SCORE BSAG INCONSEQUENTIAL BEHAVIOR	11	No	Developmental attributes
NervSymptoms	TOT SCORE BSAG MISC NERVOUS SYMPTOMS	11	No	Developmental attributes
GeneralKnowTR	CHILD'S GEN KNOWLDGE-TEACHER'S RATING	11	No	Education and health literacy
NumberTR	CHILD'S NUMBER WORK-TEACHER'S RATING	11	No	Education and health literacy
BookTR	CHILD'S USE BOOKS-TEACHER'S RATING	11	No	Education and health literacy
OralAbTR	CHILD'S ORAL ABILITY-TEACHER'S RATING	11	No	Education and health literacy
GraspEng	IMPERFECT GRASP OF ENGLISH	11	No	Education and health literacy
GeneralAbT	TOTAL SCORE ON GENERAL ABILITY TEST	11	No	Education and health literacy
ReadingCompT	READING COMPREHENSION TEST SCORE	11	No	Education and health literacy
MathT	MATHEMATICS TEST SCORE	11	No	Education and health literacy
CopyT	COPYING DESIGNS TEST SCORE	11	No	Education and health literacy
Sex	0-3D SEX OF CHILD	11	No	Demographic
Region	REGION AT NCDS2 (1969) - 11 YEARS	11	No	Demographic
HHNumber	NUMBER LIVING IN CHILD'S HOUSEHOLD	11	No	Demographic
MotherEthnicity	AREA OF WORLD IN WHICH MOTHER BORN	11	No	Demographic
FatherEthnicity	AREA OF WORLD IN WHICH FATHER BORN	11	No	Demographic
LangHome	IS ENGLISH USUALLY SPOKEN AT HOME	11	No	Demographic
FamilyMoves	NO. OF FAMILY MOVES SINCE CHLDS BIRTH	11	No	Demographic

ParentalSep	PARENTS SEPARATED	11	No	Demographic/ACE
ParentalDeath	PARENTAL DEATH	11	No	Demographic/ACE
MotherChronicCon	WHAT IS MUMS CHRONIC CONDITION M:C1-2	11	No	Transgenerational impact of parent health and health behaviours
FatherChronicCon	WHAT IS DADS CHRONIC CONDITION M:C1-2	11	No	Transgenerational impact of parent health and health behaviours
FatherWeight	FATHER'S WEIGHT IN STONES	11	No	Transgenerational impact of parent health and health behaviours
FatherHeight	FATHER'S HEIGHT IN INCHES	11	No	Transgenerational impact of parent health and health behaviours
MotherWeight	MOTHER'S WEIGHT IN STONES	11	No	Transgenerational impact of parent health and health behaviours
FatherWeight	MOTHERS HEIGHT IN INCHES	11	No	Transgenerational impact of parent health and health behaviours
HHnum	NUMBER LIVING IN CHILD'S HOUSEHOLD	11	No	Socioeconomics/Demographic
HousingTenure	TENURE OF ACCOMODATION	11	No	Socioeconomics/Demographic
NoShareBedroom	HOW MANY PEOPLE SHARE CHILDS BEDROOM	11	No	Socioeconomics/Demographic
HHAmenities	ACCESS TO HOUSEHOLD AMENITIES	11	No	Socioeconomics/Demographic
NumberPerRoom	NUMBER OF PERSONS PER ROOM	11	No	Socioeconomics/Demographic
FatherSC	SOCIAL CLASS OF FATHER OR MALE HEAD (GRO 1966)	11	No	Socioeconomics/Demographic
FatherUnemploy	FATHER,MALE HEAD'S OCCUPATION	11	No	Socioeconomics/Demographic
MotherRecSEG	MOTHERS'S MOST RECENT WORK AND SEG (GRO 1966)	11	No	Socioeconomics/Demographic
FreeSchoolMeals	DOES ANY CHILD GET FREE SCHOOL MEALS	11	No	Socioeconomics/Demographic
FinancialHardship	SERIOUS FINANCIAL HARDSHIP LAST YR	11	No	Socioeconomics/Demographic
MotherFig	CHILD'S MOTHER FIGURE	11	No	Demographic/ACE
FatherFig	CHILD'S FATHER FIGURE	11	No	Demographic/ACE
LACare	HAS CHILD EVER BEEN IN LA CARE	11	No	ACE
VolServCare	HAS CHILD BEEN IN VOL SERVICE CARE	11	No	ACE
ChildDisob	IS CHILD DISOBEDIENT AT HOME	11	No	Developmental attributes/Parental family factors and parental ability to care for child
ParSchLeaveAge	PARENTAL HOPES CHILD'S SCHOOL LEAVING	11	No	Parental family factors and parental ability to care for child
ParFurtherEd	PARS WANT FURTHR EDUC,TRAIN FOR CHLD	11	No	Parental family factors and parental ability to care for child

MotherWalk	DOES MUM TAKE CHILD FOR WALKS,VISITS	11	No	Parental family factors and parental ability to care for child
FatherWalk	DOES DAD TAKE CHILD FOR WALKS,VISITS	11	No	Parental family factors and parental ability to care for child
FatherMangChild	DADS ROLE IN MANAGEMENT OF CHILD	11	No	Parental family factors and parental ability to care for child
FatherIntrEd	FATHERS' INTEREST IN CHILDS EDUCATION	11	No	Parental family factors and parental ability to care for child
MotherIntrEd	MOTHERS' INTEREST IN CHILDS EDUCATION	11	No	Parental family factors and parental ability to care for child
PlayAreas	IS MUM HAPPY WITH PLAY AREAS NEARBY	11	No	Neighbourhood, physical environments and health care systems
PublicParks	USE OF PUBLIC PARKS, ETC IN LAST 12M	11	No	Neighbourhood, physical environments and health care systems
RecreationGround	USE OF RECREATION GRND, ETC LAST 12M	11	No	Neighbourhood, physical environments and health care systems
Overeating7	OVEREATING-REPORTED BY MOTHER	7	Yes	Health behaviours and diet
ContactProbation7	FAMILY CONTACT-PROBATION OFFICER	7	Yes	ACE
FamDiffMentalIll7	FAM DIFFICLTS-MENTAL ILLNESS,NEUROSIS	7	Yes	ACE/Transgenerational impact of parent health and health behaviours
FamDiffMentaSub7	FAM DIFFICULTIES-MENTAL SUBNORMALITY	7	Yes	ACE/Transgenerational impact of parent health and health behaviours
FamDiffDomesticTen7	FAMILY DIFFICULTIES-DOMESTIC TENSION	7	Yes	ACE
FamDiffAlcohol7	FAMILY DIFFICULTIES-ALCOHOLISM	7	Yes	ACE/Transgenerational impact of parent health and health behaviours
MotherSmoking16	NO. OF CIGARETTES MUM SMOKES PER DAY	16	Yes	Transgenerational impact of parent health and health behaviours
FatherSmoking16	NO. OF CIGARETTES DAD SMOKES PER DAY	16	Yes	Transgenerational impact of parent health and health behaviours
AnyEatingDiff7	IS THERE ANY EATING DIFFICULTY	7	Yes	Health behaviours and diet
TypeEatDisorder7	TYPE OF EATING DIFFICULTY M:C 1-2	7	Yes	Health behaviours and diet
MotherIllness16	MOTHER-DIAGNOSIS OF ILLNESS	16	Yes	Transgenerational impact of parent health and health behaviours
FatherIllness16	FATHER-DIAGNOSIS OF ILLNESS	16	Yes	Transgenerational impact of parent health and health behaviours
AptitudeSport16	APTITUDE FOR SPORTS & GAMES-STDY CHLD	16	Yes	Health behaviours and diet

SatisfyPlaceMeet16	SATISFACTION - PLACES TO MEET IN AREA	16	Yes	Neighbourhood, physical environments and health care systems
SatisfySportFac16	SATISFIED-SPORTING FACILITIES IN AREA	16	Yes	Neighbourhood, physical environments and health care systems
NoCigsAWeek16	NO. OF CIGARETTES SMOKED PER WEEK	16	Yes	Health behaviours and diet
HowLongSinceAlcohol16	HOW LONG SINCE CHILD DRANK ALCOHOL	16	Yes	Health behaviours and diet
AlcoholLastWeek16	NO. AND TYPE DRINKS LAST WEEK-MC 1:3	16	Yes	Health behaviours and diet
Childactive7	CHILD NORMALLY ACTIVE-MUMS VIEW	16	Yes	Health behaviours and diet
Sport11	PUPIL TAKES PART IN SPORT OUT OF SCHOOL	11	No	Health behaviours and diet
Outdoorsport16	HOW OFTEN PLAYS OUTDOOR GAMES & SPORT	16	Yes	Health behaviours and diet
Indoorsport16	HOW OFTEN PLAYS INDOOR GAMES & SPORT	16	Yes	Health behaviours and diet

All variables selected from the data audit in the NCDS at birth, age 7, age 11 and age 16

Supplementary Table 3. Variables identified from the data audit in ACONF

Variable	Description	Sweep of data collection	Supplementary variable	Domain
Rs001	Sex	Reading survey	N/A	Demographic
M_matage	Maternal age	Reading survey	N/A	Demographic
Rs004	Place of birth	Reading survey	N/A	Demographic
Rs007	Population size	Reading survey	N/A	Demographic
M_numsib	Number of siblings	Reading survey	N/A	Demographic
Rs035	Family size	Reading survey	N/A	Demographic
Rs041	Marital status at birth	Reading survey	N/A	Demographic
Fs101	Any step children	Family survey	N/A	Demographic
Rs008	Mobility	Reading survey	N/A	Socioeconomic
Rs009	Class grade	Reading survey	N/A	Socioeconomic
Rs014	Number of schools attended	Reading survey	N/A	Socioeconomic
Rs027	Area of residence	Reading survey	N/A	Socioeconomic
Rs032	Father social class	Reading survey	N/A	Socioeconomic
Rs041	Mother occupation	Reading survey	N/A	Socioeconomic
Rs042	Wife education	Reading survey	N/A	Socioeconomic

Fs106	Mother school leaving age	Family survey	N/A	Socioeconomic
Sch_mate	Mother further education	Family survey	N/A	Socioeconomic
Fs202	Housing tenure	Family survey	N/A	Socioeconomic
Fs203	Number of rooms	Family survey	N/A	Socioeconomic
Fs212	Household size	Family survey	N/A	Socioeconomic
Fs374	Father unemployment	Family survey	N/A	Socioeconomic
C_phhcon	Percentage renting from council	Reading survey	N/A	Neighbourhood, physical environments and health care systems
C_phhown	Percentage of owner-occupied houses	Reading survey	N/A	Neighbourhood, physical environments and health care systems
C_phhhtn	Percentage of houses with hot water	Reading survey	N/A	Neighbourhood, physical environments and health care systems
C_phcldn	Percentage of houses with cold water	Reading survey	N/A	Neighbourhood, physical environments and health care systems
C_phbthn	Percentage of households with no fixed bath	Reading survey	N/A	Neighbourhood, physical environments and health care systems
C_phhwc	Percentage of households with shared WC	Reading survey	N/A	Neighbourhood, physical environments and health care systems
Fs054	Behavioural problems	Family survey	N/A	Developmental attributes
Fs135	Rutter scale A	Family survey	N/A	Developmental attributes
Fs219	Sociability of child	Family survey	N/A	Developmental attributes
Rs173	Anti-social score scale B	Reading survey	N/A	Developmental attributes
Rs174	Neurotic score scale B	Reading survey	N/A	Developmental attributes
Rs175	Total score scale B	Reading survey	N/A	Developmental attributes
Rs176	Neurotic/anti-social rating	Reading survey	N/A	Developmental attributes

Rs030	Absences from school for health reasons	Reading survey	N/A	Child health including check-ups and screening
Rs109	Number of medical exams	Reading survey	N/A	Child health including check-ups and screening
Rs113-rs115	Height	Reading survey	N/A	Child health including check-ups and screening
Rs117-rs118	Weight	Reading survey	N/A	Child health including check-ups and screening
Fs015	Hospital admissions	Family survey	N/A	Child health including check-ups and screening
Fs025	Asthma	Family survey	N/A	Child health including check-ups and screening
Fs026	Measles	Family survey	N/A	Child health including check-ups and screening
Fs027	Hay fever	Family survey	N/A	Child health including check-ups and screening
Fs028	Whooping cough	Family survey	N/A	Child health including check-ups and screening
Fs029	Eczema	Family survey	N/A	Child health including check-ups and screening
Fs030	Convulsions/fits	Family survey	N/A	Child health including check-ups and screening
Fs031	Meningitis	Family survey	N/A	Child health including check-ups and screening
Fs032	Cold	Family survey	N/A	Child health including check-ups and screening
Fs033	Sore throat	Family survey	N/A	Child health including check-ups and screening
Fs034	Cough	Family survey	N/A	Child health including check-ups and screening
Fs035	Bronchitis	Family survey	N/A	Child health including check-ups and screening
Fs036	Hives	Family survey	N/A	Child health including check-ups and screening
Fs037	Stomach ache	Family survey	N/A	Child health including check-ups and screening

Fs038	Ear ache	Family survey	N/A	Child health including check-ups and screening
Fs039	Other frequent illness	Family survey	N/A	Child health including check-ups and screening
Fs040	Shortness of breath	Family survey	N/A	Child health including check-ups and screening
Fs041	Wheezy chest	Family survey	N/A	Child health including check-ups and screening
Fs051	Physical health	Family survey	N/A	Child health including check-ups and screening
Fs052	Mental and emotional health	Family survey	N/A	Child health including check-ups and screening
Fs073	Nursery school attendance	Family survey	N/A	Education and health literacy
Rs180	Intelligence test at age 7	Reading survey	N/A	Education and health literacy
Rs181	Intelligence test at age 9	Reading survey	N/A	Education and health literacy
Rs094	Type of school	Reading survey	N/A	Education and health literacy
Rs179	Degree of over/under achievement at age 11	Reading survey	N/A	Education and health literacy
Sch_iq7	School IQ at age 7	Family survey	N/A	Education and health literacy
Sch_iq9	School IQ at age 9	Reading survey	N/A	Education and health literacy
Rs074	Mother physical grade	Reading survey	N/A	Prenatal, antenatal, neonatal and birth
Rs090	Child physical grade	Reading survey	N/A	Prenatal, antenatal, neonatal and birth
Rs076	Haemorrhage complications	Reading survey	N/A	Prenatal, antenatal, neonatal and birth
Rs080	Type of delivery	Reading survey	N/A	Prenatal, antenatal, neonatal and birth
Rs081	Length of labour	Reading survey	N/A	Prenatal, antenatal, neonatal and birth

Rs082	Caesarean	Reading survey	N/A	Prenatal, antenatal, neonatal and birth
Rs083-rs084	Week of antenatal visit	Reading survey	N/A	Prenatal, antenatal, neonatal and birth
Rs085-rs086	Gestational length	Reading survey	N/A	Prenatal, antenatal, neonatal and birth
Rs087	Birth weight	Reading survey	N/A	Prenatal, antenatal, neonatal and birth
Rs075	Pre eclamptic toxaemia complications	Reading survey	N/A	Prenatal, antenatal, neonatal and birth

All variables selected from the data audit in the ACONF at ages 6-12.

Supplementary Figure 1. PCA analysis mapping mutually exclusive variables within each domain based on similar characteristics in ACONF.

PDF Document

Supplementary Figure 2. Using PCA analysis to map mutually exclusive variables within each domain based on similar characteristics in NCDS.

PDF Document

Supplementary Figure 3. Using PCA analysis to map mutually exclusive variables within each domain based on similar characteristics in NCDS.

PDF Document
